# Supplementary figures and images for: Alteration of Colonic Bacterial and Fungal Composition and Their Inter- and Intra-Kingdom Interaction in Patients with Adenomas with Low-Grade Dysplasia
Source: Microorganisms. 2023 May 18;11(5):1327. doi: 10.3390/microorganisms11051327 (PMC10223777; doi:10.3390/microorganisms11051327)

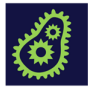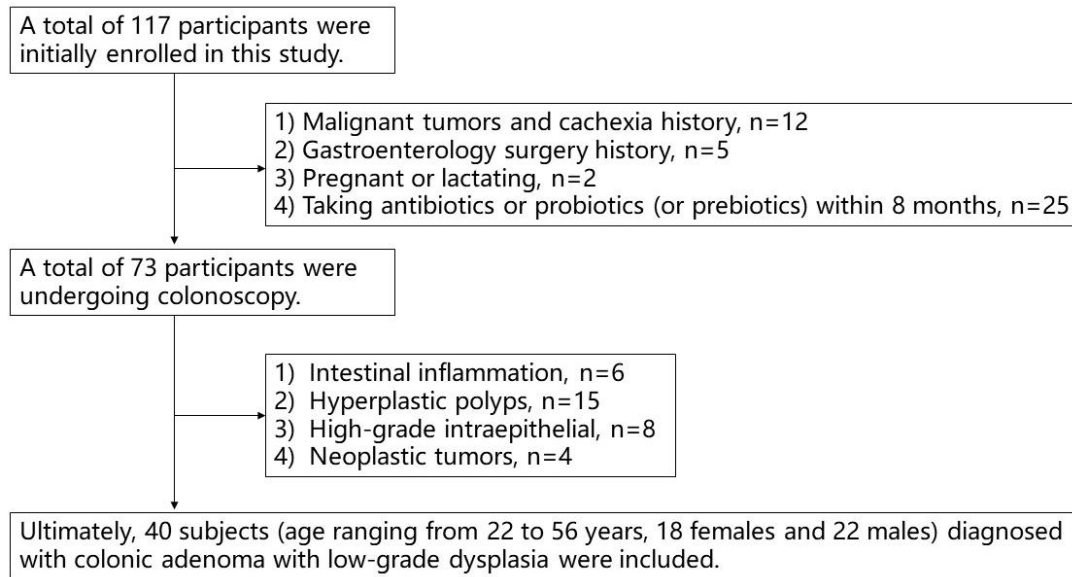

Supplementary Figure S1. Flowchart of participant selection.

Supplement: Supplementary file 1 [file microorganisms-11-01327-s001.zip › Supplementary Figure S1 microorganisms-2320648-supplementary.docx.pdf]
